# Alcoholic pontine myelinolysis: beware the stroke mimic

**DOI:** 10.1259/bjrcr.20210005

**Published:** 2021-03-23

**Authors:** Kieran Kusel, Omar Azzam, Adam Youssef, David Prentice

**Affiliations:** 1 Department of Radiology, Royal Perth Hospital, Perth, WA, Australia; 2 Department of Internal Medicine, Royal Perth Hospital, Perth, WA, Australia; 3 Neurological Intervention and Imaging Service of Western Australia, Royal Perth Hospital, Perth, WA, Australia; 4 Perron Institute for Neurological and Translational Science, Nedlands, WA, Australia

## Abstract

Central pontine myelinolysis (CPM), often referred to as osmotic demyelination syndrome, is most commonly seen in the setting of rapid correction of hyponatraemia. Although imaging is the key to diagnosis, conventional CT and MRI findings often lag the clinical manifestations and characteristic MRI changes may be delayed by up to 14 days.

We present a case of a 45-year-old female with an extensive history of alcohol misuse and malnutrition who presented with left hemiparesis, initially suspected to be a stroke. This was following a recent hospital admission when she was managed for Wernicke’s encephalopathy and treated with electrolyte and vitamin replacement. As part of a “code stroke” protocol, CT was initially performed. The initial non-contrast CT brain and CT angiogram of the intracranial arteries were normal, but a CT brain perfusion study demonstrated increased pontine blood flow. A subsequent MRI of the brain confirmed CPM, which was congruent with her clinical course.

This case highlights the importance of osmotic demyelination as a stroke mimic. CPM should be considered in alcoholic patients with neurological impairment regardless of serum sodium. To our knowledge, this is the first published case which illustrates CT perfusion changes in CPM. MRI, however, remains essential for diagnosis.

## Case report

A 45-year-old female with a history of chronic alcohol misuse presented to our service with acute onset left hemiparesis and drowsiness. This was seven days post-discharge from an earlier hospital admission when she was treated for Wernicke’s encephalopathy with high-dose intravenous thiamine, electrolyte replacement and vitamin D. During this previous admission, her serum sodium concentration was corrected from 127 to 137 mmol l^−1^ over a five-day period. She received high-dose intravenous thiamine therapy with good improvement in gait and was discharged home on oral thiamine, multivitamin and vitamin D supplementation.

**Figure 1.**
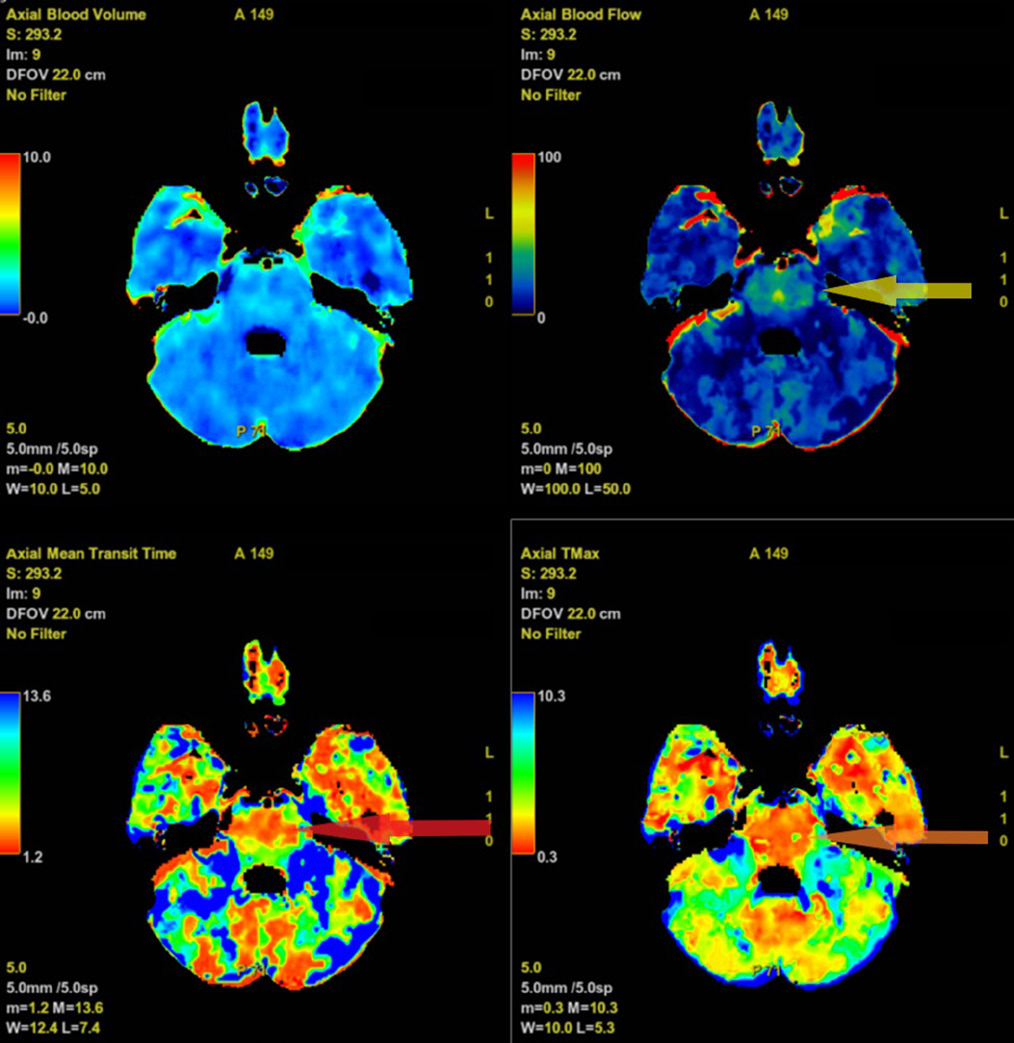
CT perfusion images at the level of the pons. This demonstrates increased blood flow (top right, yellow arrow), reduced mean transit time (bottom left, red arrow) and reduced time-to-maximum (bottom right, orange arrow) in the upper pons.

**Figure 2.**
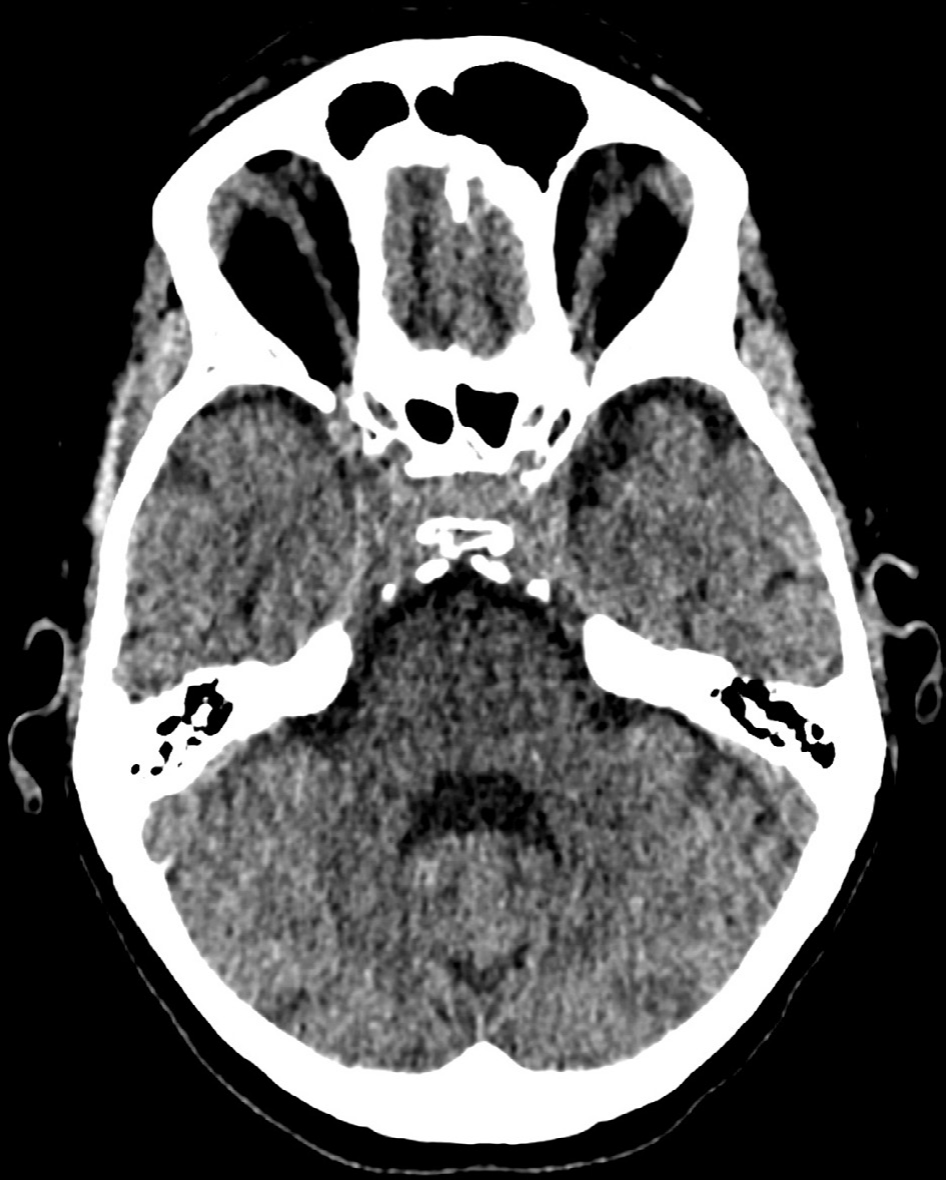
Axial, non-contrast CT image at the same level, at the level of the pons.

On the subsequent presentation seven days later, she presented with acute onset left hemiparesis and drowsiness. Given the acute onset of her symptoms, she was promptly investigated with a non-contrast CT head, CT angiogram of the intracranial arteries and CT brain perfusion as part of a “code stroke” protocol. The non-contrast CT head and CT angiogram were normal. CT perfusion demonstrated increased blood flow to the pons with corresponding reduction in mean transit time and time-to-maximum (Tmax) (Figures 1 and 2).

**Figure 3.**
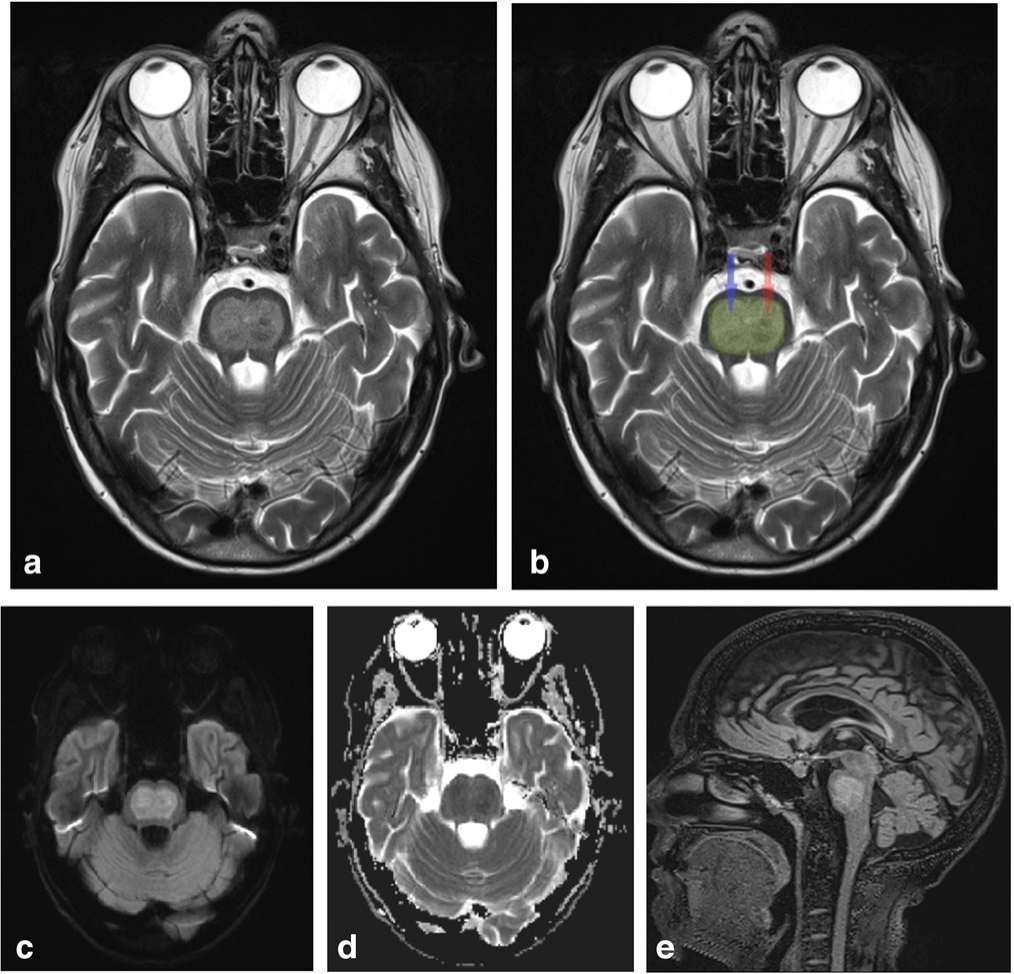
a: Axial T2W image demonstrates high T2-signal intensity in the upper pons with resemblance to a pig’s snout (the so-called “piglet sign”). b: The same axial T2W image with the area of oedema of the pons highlighted in green. The transverse pontine fibres (pontocerebellar fibres and median raphe) are most affected and there is sparing of the peripheral fibres (ventrolateral longitudinal fibres) and corticospinal tracts, giving rise to the pig’s snout appearance. Notably in this case, oedema involves the right corticospinal tracts (blue arrow), which explains why the patient had left-sided hemiparesis (this is above the level of decussation of corticospinal tracts, with 90% of tracts crossing the midline at the level of the medulla). c and d: DWI and ADC map show corresponding restricted diffusion in the pons. This is typically the earliest MRI finding in CPM. e: Sagittal FLAIR image demonstrates increased FLAIR signal in the pons.

She was admitted under neurology and her symptoms persisted. An MRI of the brain was performed on day two of her admission. This demonstrated restricted diffusion within the pons with corresponding increased signal intensity on T2W and fluid-attenuated inversion recovery (FLAIR) sequences (Figure 3). The high T2 and FLAIR signal in the upper pons on axial images resembled a pig’s snout (the so-called “piglet sign” in CPM), with relative sparing of peripheral fibres (ventrolateral longitudinal fibres) and corticospinal tracts (also classically described as a trident shaped appearance). In this case, however, the right corticospinal tracts were also involved (the right nostril of the pig’s snout), which may explain why she had developed left-sided hemiplegia. There was no imaging evidence for extrapontine myelinolysis.

## Discussion

Alcohol dependence is associated with neurological disorders such as Wernicke’s encephalopathy, traumatic brain injury, stroke, withdrawal seizures, Marchiafava-Bignami disease and cerebellar degeneration. Osmotic demyelination syndrome is usually not considered in the absence of hyponatremia and may be easily overlooked if features such as neurobehavioural disorders, confusion, drowsiness and ataxia are attributed to other conditions. Imaging is the key to the diagnosis but the characteristic MRI changes may be delayed by up to 14 days. Post-mortem incidence of unsuspected osmotic demyelination is 0.5%.^1^

Clinical features of osmotic demyelination syndrome include quadriparesis, locked in syndrome and coma. Rarely, patients may develop ophthalmoplegia if there is involvement of the pontine tegmentum.^2^ Other less specific signs include lethargy, confusion and neurobehavioral changes (such as acute psychosis, catatonia and emotional lability), which can be easily mistaken for delirium in these patients. Extrapontine demyelination is often found in the basal ganglia with clinical features of parkinsonism, movement disorder (chorea, myoclonus) and dysarthria.^3^

In this case, the patient had been treated for Wernicke’s encephalopathy during a recent, earlier admission to hospital. During this earlier admission, her hyponatraemia was corrected over a five-day period when her serum sodium increased from 127 to 137 mmol l^−1^. She also received intravenous thiamine, vitamin D and other electrolyte replacement. When she represented to hospital seven days later, she had developed acute onset left hemiparesis and stroke was initially suspected. This case highlights the importance of considering osmotic demyelination syndrome in high-risk patients who present with stroke-like symptoms.

While MR perfusion changes have been reported in CPM, to our knowledge this is the only case report which demonstrates the changes of CPM on CT brain perfusion.^4^ CT perfusion demonstrated increased blood flow, reduced mean transit time and reduced time-to-maximum in the pons, which may be due to the increased metabolic demands at the site of cell damage. MRI confirmed the diagnosis of osmotic demyelination and demonstrated diffusion restriction, T2 hyperintensity and T1 hypointensity in the upper pons with a typical “piglet sign” pattern. Notably in this case, the right corticospinal tracts were affected, which may explain why the patient had developed left hemiparesis.

### CPM and Wernicke’s encephalopathy

Concurrent osmotic demyelination and Wernicke’s encephalopathy have been described in a handful of cases.^5–7^ Three of these cases were in young females with hyperemesis gravidarum in which demyelination occurred in the setting of mild hyponatremia and followed treatment for Wernicke’s encephalopathy.

This suggests that malnutrition, and in particular the depletion of water-soluble vitamins, may contribute to the pathogenesis of osmotic demyelination syndrome. One hypothesis is that thiamine or other vitamin deficiencies prime the pontocerebellar fibres susceptibility to demyelination. Both the basal ganglia and the pons contain compacted interlaced highly myelinated white matter and grey matter nuclei, which have high metabolic requirements. Oligodendrocytes, the glial cells of the central nervous system, which produce the myelin sheath, are very sensitive to hypoxia and are a major supply of ATP to axons through lactate transport. It is conceivable that malnutrition and loss of electrolytes (potassium and phosphate) would interfere with Na^+^/K^+^ ATPase and water channel pumps (aquaporins).^8^ Oligodendrocytes also contain iron which if released by demyelination may be toxic in its ferrous form.

The most common cause of osmotic demyelination is the rapid correction of hyponatremia in patients with chronic hyponatremia. Rapid overcorrection is especially likely in situations of psychogenic polydipsia, vasopressin use and beer potomania where solute replacement leads to substantial free water clearance. Another water-soluble vitamin deficiency in alcoholics is ascorbic acid. Interestingly, ascorbic acid transport into neurones requires a sodium-dependent transporter (SVCT2). Low sodium and other cations will reduce ascorbic acid transport and thus increase oxidative damage.^9^

### Imaging findings in CPM

**Figure 4.**
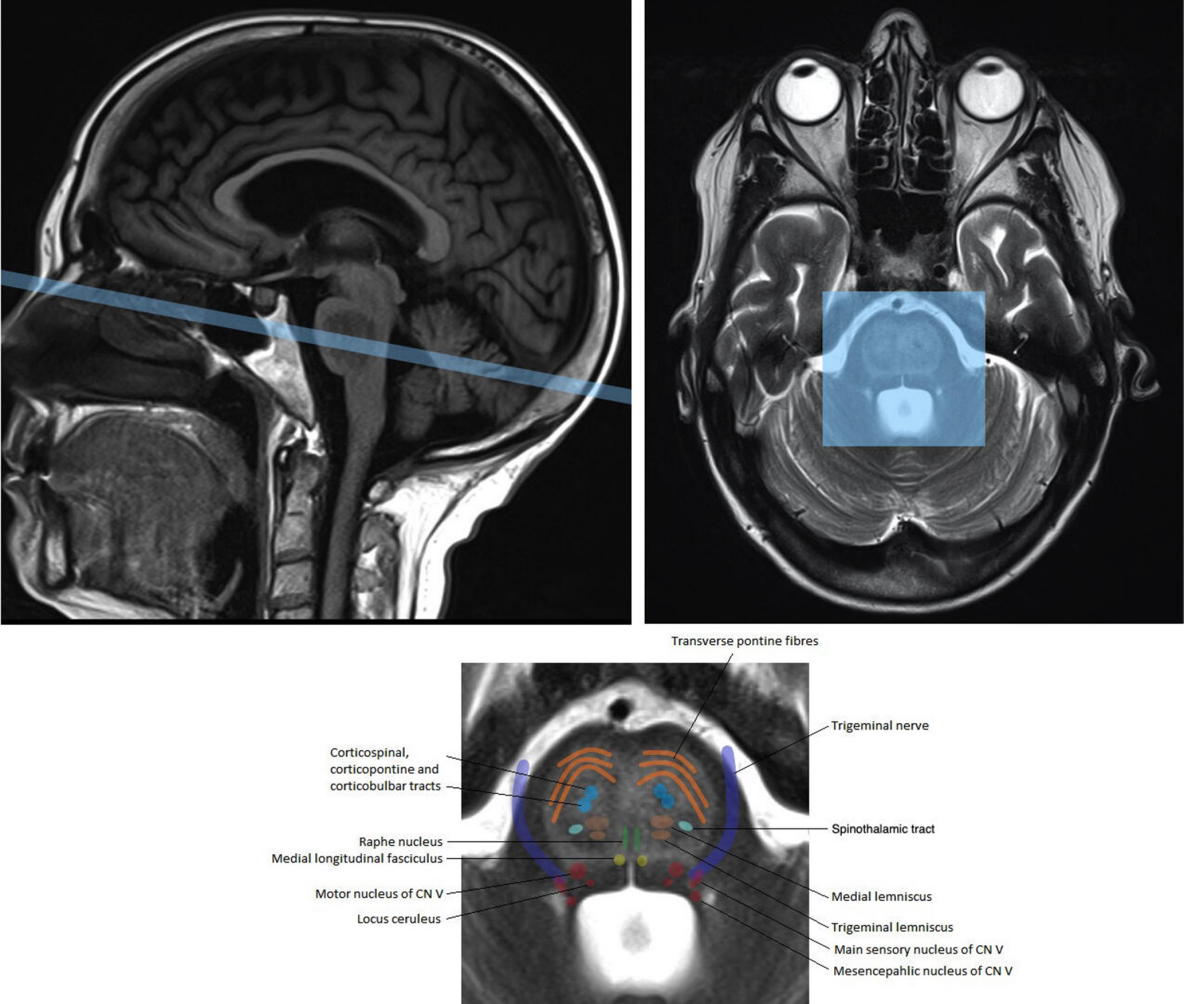
Labelled axial image demonstrating the position of the major tracts and fibres within the pons. The corticospinal tracts (highlighted in light blue) are often spared in central pontine myelinolysis giving rise to the nostrils of the pig’s snout in the so-called piglet sign.

Although imaging is key to diagnosis, conventional CT and MRI findings often lag behind the clinical manifestations of CPM. Diffusion restriction on MR is usually the first change seen which may be present within 24 hours of onset of symptoms but has no prognostic significance.^10^ High signal intensity on T2W or FLAIR images and low signal intensity on T1W images are often seen in the later stages and this is due to vasogenic oedema in the pontocerebellar white matter. The high signal on axial T2W images is typically described as being shaped like a trident (trident sign) or the snout of a pig (piglet sign). This is due to vasogenic oedema of the white matter of the pontocerebellar fibres and median raphe with relative sparing of the descending corticospinal and corticobulbar tracts (the nostrils of the pig snout) (Figure 4). If the pontine tegmentum is involved, ocular abnormalities and coma may occur. Rare involvement of the medial lemniscus causes sensory ataxia. The unusual topography of the lesions suggests hypoxia or arterial ischaemia with oedema compromising blood flow. It seems that the paramedian pontine arterial territory is involved as it is in basilar arterial disease and hypertensive small vessel disease. The sudden hemiparesis in our case could be due to transient ischaemia of the corticospinal tracts consequent on pontine oedema.

A PET scan in week two may demonstrate avid FDG uptake in the pons as a result of microglia inflammation.^11^ This also explains why increased blood flow and reduced mean transit time was seen on our CT perfusion study.

### Management of CPM

Management of osmotic demyelination is mainly supportive. Intravenous high-dose steroids have been successful in a small number of patients in reversing osmotic demyelination by enhancing the blood-brain barrier.^12,13^ In a single case, early reduction of the overcorrected sodium led to the reversal of the demyelination.^14^ Other trialled treatments have included plasmapheresis (on the basis of inflammatory response),^15^ pramipexole, IVIG, minocycline^16^ and thyroid releasing hormone,^17^ but with variable outcomes.

Historically, osmotic demyelination was associated with a high mortality rate in the order of 90%, but more recent figures are closer to 30%.^18^ Long-term outcomes for survivors in a hospitalised intensive care unit French cohort were: 33–50% living independently but 30% requiring ongoing supportive therapy. A normonatraemic variant (*i.e.* with no correction phase of hyponatraemia) of CPM in alcoholism that tends to have benign outcomes has been described in a Japanese cohort.^19^

## Conclusion

Osmotic demyelination needs to be considered in alcoholic patients with neurological impairment regardless of serum sodium. Replacement of electrolytes and vitamins may prevent demyelination or limit it. This case highlights the importance of osmotic demyelination as a stroke mimic. To our knowledge, this is the first published case that illustrates CT perfusion changes in central pontine myelinolysis. MRI is an essential tool for the diagnosis as the symptoms and signs may be non-specific. Death and severe disability are not uncommon.

## Learning points

1. Central pontine myelinolysis or osmotic demyelination syndrome is most commonly seen in the setting of rapid correction of hyponatraemia.
2. It should be considered in alcoholic and malnourished patients with vitamin deficiencies and neurological impairment regardless of serum sodium. In these patients, it may be easily overlooked when neurological signs and symptoms are attributed to delirium or other conditions.
3. Imaging is essential for diagnosis, however, CT and MRI findings often lag behind the clinical manifestations.
4. This case highlights the importance of osmotic demyelination as a stroke mimic and illustrates the changes seen on CT perfusion and MRI.
